# Controlled-Release Materials for Remediation of Trichloroethylene Contamination in Groundwater

**DOI:** 10.3390/ma16217045

**Published:** 2023-11-05

**Authors:** Shan Zhao, Jianhua Wang, Wenjin Zhu

**Affiliations:** 1 College of Ocean Science and Engineering, Shanghai Maritime University, Shanghai 201306, China; 18805488595@163.com; 2 College of Civil Engineering, Tongji University, Shanghai 200092, China; 3 School of Civil and Ocean Engineering, Jiangsu Ocean University, Lianyungang 222005, China

**Keywords:** controlled-release materials, trichloroethylene, oxidant-based, reductant, electron donor, groundwater

## Abstract

Groundwater contamination by trichloroethylene (TCE) presents a pressing environmental challenge with far-reaching consequences. Traditional remediation methods have shown limitations in effectively addressing TCE contamination. This study reviews the limitations of conventional remediation techniques and investigates the application of oxidant-based controlled-release materials, including encapsulated, loaded, and gel-based potassium permanganate since the year 2000. Additionally, it examines reductant controlled-release materials and electron donor-release materials such as tetrabutyl orthosilicate (TBOS) and polyhydroxybutyrate (PHB). The findings suggest that controlled-release materials offer a promising avenue for enhancing TCE degradation and promoting groundwater restoration. This study concludes by highlighting the future research directions and the potential of controlled-release materials in addressing TCE contamination challenges.

## 1. Introduction 

Groundwater is an essential component of the Earth’s hydroecological system. It plays a pivotal role in ensuring energy and food security, safeguarding human health, and maintaining ecological stability, making it an important strategic resource [1,2]. Currently, groundwater serves as the primary water source for approximately half of the global population [3]. Most rural areas in both developed and developing countries depend solely on groundwater for drinking, such as China [4]. However, anthropogenic activities and the impacts of climate change have disrupted the delicate balance, resulting in groundwater overexploitation and the depletion of this invaluable resource [5]. Furthermore, with the continuous development of industry and agriculture, an increasing number of chemical raw materials are being utilized in people’s production and daily life, leading to an unsatisfactory state of groundwater safety. The 2022 China’s Ecological and Environmental Condition Report underscores the alarming state of groundwater quality, with 22.4% of assessed points falling into Class V, indicating severe contamination [6]. Among the culprits, trichloroethylene (TCE), a prominent chlorinated organic pollutant widely used in industrial processes, solvent applications, and potential sources of contamination, poses a significant threat to groundwater ecosystems.

TCE has already been extensively used as a significant industrial chemical in various sectors including agriculture [7], industry [8], and medicine [9] since the 1960s, leading to comprehensive toxicological investigations in subsequent decades. These studies unveiled its potential health hazards, affecting vital organs such as the nervous system, heart, liver, and kidneys [10,11]. In 2002, both the United States Environmental Protection Agency and the International Agency for Research on Cancer classified TCE as a carcinogen [12,13]. The 2022 US Environmental Protection Agency’s draft of the Toxic Substances Control Act risk assessment further underscores TCE’s human health risks [14].

TCE contamination sources fall into two primary categories: point source, i.e., a single, identifiable source of pollution that can be traced back to a specific location, and non-point source, i.e., more diffuse, multiple, and dispersed sources for which it is challenging to pinpoint the exact origin of contamination. Non-point source pollution, characterized by diffuse and challenging-to-monitor emissions, silently infiltrates environments, posing imperceptible exposure risks [15,16,17]. Due to the limited self-regulation capacity of ecosystems, achieving harmless levels of TCE in groundwater through natural degradation alone would require an extended timeframe. Once the level of contamination exceeds the self-regulatory limits of the ecosystem, it might even lead to an imbalance in the entire ecological system. Consequently, various artificial intervention methods have emerged for remediation. Currently, many experts and scholars have proposed numerous viable solutions for the restoration and management of TCE. Some of these solutions, such as in situ bioremediation technology [18], permeable reactive barrier (PRB) remediation technology [19], and in situ chemical oxidation technology [20], have been effectively applied in site remediation processes. However, due to TCE’s characteristics as a heavy non-aqueous phase liquid, with fast migration, the potential for retention, resistance to degradation, and high toxicity, it is prone to issues such as pollutant rebound. Therefore, the efficient treatment of TCE in groundwater remains one of the key and challenging aspects of current research on groundwater remediation technologies.

Since the 1940s, some scholars in the United States initially researched the preparation and properties of urea–formaldehyde controlled-release fertilizers, applying these slow-release materials to the field of agriculture [21,22]. Starting from the 1990s, oral controlled-release formulations have become a significant research focus in the pharmaceutical field due to their mechanism of once-daily dosing, controlled, targeted, and timed release [23,24]. Currently, controlled-release materials have found widespread use in both the agricultural [25] and pharmaceutical [26] fields. However, due to factors such as site complexity [27,28] and economic considerations, research on controlled-release materials in the domain of groundwater pollution control has predominantly remained at the level of laboratory theoretical studies. They have not yet been extensively applied in the remediation processes of groundwater pollution sites. Therefore, this study begins by reviewing the traditional remediation methods employed for TCE-contaminated groundwater and elucidates their limitations. Subsequently, an exploration of the different types of delivery materials and their respective mechanisms of action is conducted. Through a comprehensive exploration of case studies and real-world applications, the successes and challenges associated with these materials are highlighted. Furthermore, the environmental and ethical considerations surrounding their use are discussed. Finally, the future trends and research directions in the field of TCE remediation using delivery materials are outlined. As TCE contamination continues to threaten the integrity of groundwater resources, the utilization of delivery materials represents a promising avenue toward more effective and sustainable remediation strategies.

## 2. Traditional Remediation Methods and Their Limitations

Several traditional methods have been employed for the remediation of groundwater contaminated with TCE, each with its advantages and limitations. One of the most commonly utilized approaches is the pump-and-treat method, where groundwater is extracted from contaminated zones, treated, and then re-injected into the aquifer [29]. While this method has demonstrated some success in reducing TCE concentrations, it presents several limitations. The pump-and-treat process can be energy-intensive due to the need for extensive pumping and treatment. Additionally, it may not effectively address TCE in low-permeability zones, thus requiring prolonged treatment periods and resulting in incomplete remediation [30]. Another widely applied technique is air sparging, involving the injection of air into the saturated zone to enhance the volatilization and biodegradation of TCE [31]. However, this method may not fully address dissolved-phase TCE, particularly in low-permeability soils and aquifers. The challenge lies in effectively controlling and optimizing the distribution of injected air, leading to uneven treatment and, in some cases, the potential release of contaminants to the atmosphere [32]. Chemical oxidation methods, such as in situ oxidation, have also been employed to transform TCE into less harmful compounds. Nonetheless, the effectiveness of chemical oxidation can be constrained by the need for controlled injection and the risk of excessive reagent use, which can result in the production of harmful byproducts or incomplete treatment [33]. Bioremediation, which relies on microbial degradation of TCE and its transformation into less toxic substances, is environmentally friendly and cost-effective. However, it is often characterized by slow treatment rates, as it can be influenced by various factors including temperature, pH, and the availability of suitable electron donors [34]. Furthermore, bioremediation may struggle to treat TCE in areas with limited microbial activity or in regions with low nutrient availability [35]. Physical containment methods such as impermeable barrier walls have also been used to isolate and control the spread of TCE plumes. While these methods can be effective in certain scenarios, they are typically expensive and may not provide a permanent solution, especially in dynamic hydrogeological settings where TCE can migrate around or under barriers [36]. 

While traditional remediation methods have their merits and have been employed with varying degrees of success, they are often associated with limitations such as energy consumption, slow treatment rates, incomplete contaminant removal, and potential environmental risks (Table 1). These limitations underscore the need for innovative approaches, including the utilization of delivery materials, to address TCE contamination more comprehensively and effectively.

## 3. Application of Controlled-Release Materials

### 3.1. Concept of Controlled-Release Materials

Groundwater contamination, particularly by TCE, presents a complex environmental challenge that necessitates innovative and effective remediation strategies. Traditional remediation techniques often involve the injection of chemicals directly into contaminated aquifers. While these methods can be successful to some extent, they are not without limitations, such as the rapid depletion of active agents, the risk of secondary pollution, and the need for repeated injections. Controlled-release materials have emerged as a promising solution to overcome these limitations and enhance the efficiency of groundwater remediation efforts.

**Figure 1:**
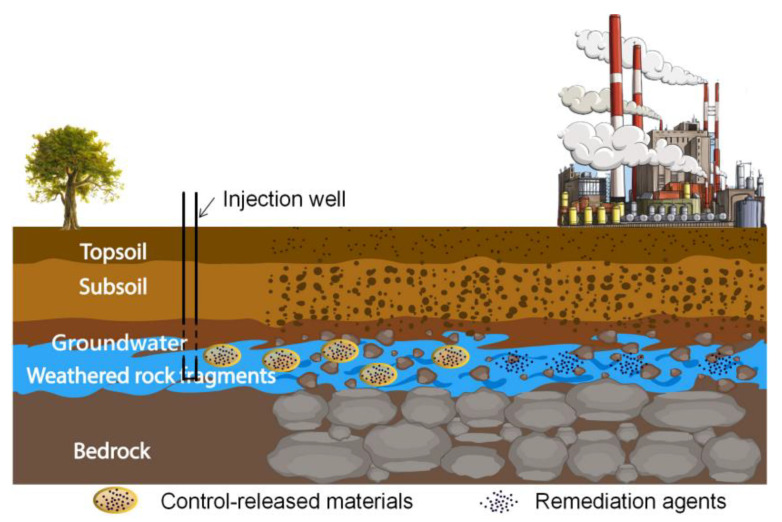
Release mechanism of controlled-release materials.

Controlled-release materials, in the context of groundwater remediation, refer to engineered substances designed to gradually release active agents over an extended period (Figure 1). These materials are tailored to address the unique challenges posed by contaminants like TCE. The controlled release of active agents, such as oxidants or reductants, allows for sustained and targeted treatment of contaminated groundwater, reducing the risk of both under-dosing and over-dosing.

Controlled-release materials come in various forms, each designed to address specific groundwater remediation challenges. The three primary categories of controlled-release materials are as follows: oxidant-based materials, reductant-based materials, and organic electron donor-based materials.

### 3.2. Oxidant-Based Controlled-Release Materials 

In the process of managing TCE groundwater pollution, traditional in situ chemical oxidation (ISCO) technology has seen widespread application due to its advantages of straightforward oxidant preparation, easy deployment, and quantitative control. Currently, a commonly used oxidant for the remediation of TCE pollution is potassium permanganate (KMnO_4_) [37]. Some researchers employed variable-controlled methods to place different concentration ratios of KMnO_4_ and TCE solutions on oscillators to conduct oxidation reactions. The results indicated a linear correlation between the oxidation reaction rate of TCE and the concentration of KMnO_4_. As the concentration of KMnO_4_ increased, the rate of pollutant removal continued to increase proportionally up to a certain threshold concentration [38]. Presently, it is generally believed that the reaction rate of TCE removal using KMnO_4_ is mainly related to the concentration of reactants, rather than being influenced by the pH value and ion strength of the contaminated site [39]. Although the oxidation of TCE using KMnO_4_ might yield products such as glyoxylic acid, glycolic acid, and oxalic acid due to the different reaction conditions, including those related to acidity and alkalinity, the overall reaction mechanism is commonly represented as follows [39]:

C_2_HCl_3_ + 2KMnO_4_ → 2MnO_2_(s) + 3Cl^−^ + H^+^ + 2CO_2_(g) + 2K^+^ (1)

According to the reaction equation and previous experimental results, it is evident that while utilizing KMnO_4_ oxidation presents advantages such as strong reactivity and ease of reaction, there are also issues that require attention and improvement: (1) KMnO_4_ can achieve effective removal within a relatively short reaction time, but for persistent pollution plumes, achieving satisfactory removal efficiency conveniently and rapidly becomes challenging; (2) during the oxidation reaction, insoluble MnO_2_ is formed from KMnO_4_, which can lead to pore clogging in soil and hinder effective contact with pollutants [40,41], thus affecting removal efficiency. Oxidant-based controlled-release materials based on KMnO_4_ have effectively addressed these issues. Taking KMnO_4_ as an example, these materials can be classified into three major categories based on their preparation methods. The following section outlines the status of preparation of three distinct types of oxidant-based controlled-release materials.

#### 3.2.1. Encapsulated KMnO_4_

**Figure 2:**
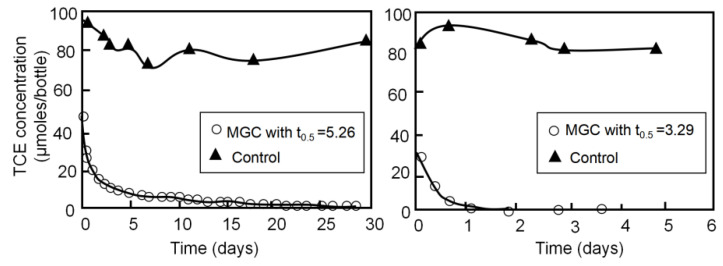
Observed TCE concentrations during batch degradation tests with microcapsules with t_0.5_ 5.26 and 3.29 (the time required to release half of C_r,max_), and during a control test with no microcapsules (revised from [42]).

Encapsulated controlled-release materials, as demonstrated in the study by Ross et al. [42], provide an effective solution to the problem of excessively rapid reactions resulting from direct contact between oxidants and reactants in environmental remediation processes. In their research, Ross and his colleagues developed two distinct types of microcapsules designed to control the release of KMnO_4_. The first type of microcapsules, referred to as single-grain-core (SGC) materials, consisted of a single KMnO_4_ core enclosed within a polymer shell composed of various waxy polymers. The second type, multiple-grain-core (MGC) materials, contained 5–10 KMnO_4_ cores encapsulated within the same polymer shell. The primary objective was to investigate and compare the release kinetics of KMnO_4_ when these microcapsules were introduced into water, and simultaneously, the researchers monitored the degradation rate of TCE (Figure 2). It was observed that the microcapsules, whether in the SCG or MCG form, exhibited a significantly prolonged release of KMnO_4_ when compared to that of non-encapsulated KMnO_4_. This extended release duration of active substances is of paramount importance in environmental remediation efforts, as it translates to a longer-lasting impact on pollutants in the water.

One of the key advantages of encapsulated KMnO_4_ is its ability to release KMnO_4_ gradually over several weeks, thereby effectively enhancing its capacity to degrade TCE and other contaminants. This controlled release mechanism allows for a more sustained and controlled approach to groundwater pollutant remediation, mitigating the risk of sudden and uncontrollable reactions. Furthermore, the protective polymer shell surrounding the KMnO_4_ within the microcapsules serves as a barrier, reducing its reactivity with other substances during transport and application. This protective layer enhances the overall utilization of KMnO_4_ and ensures that it can reach its intended target without being prematurely consumed or reacting with unintended compounds.

However, it is essential to acknowledge that the preparation process for encapsulated controlled-release materials is inherently complex and often associated with higher production costs. Additionally, there is a potential risk of secondary pollution to water bodies, stemming from the release of materials used in the microcapsule shells or any residues of the encapsulation process itself. Therefore, further research in this field should be conducted with meticulous attention to both the efficacy and the environmental implications of encapsulated controlled-release materials. A careful consideration of these factors is crucial to strike a balance between the benefits of prolonged pollutant action and the potential drawbacks associated with their application.

#### 3.2.2. Loaded KMnO_4_

**Figure 3:**
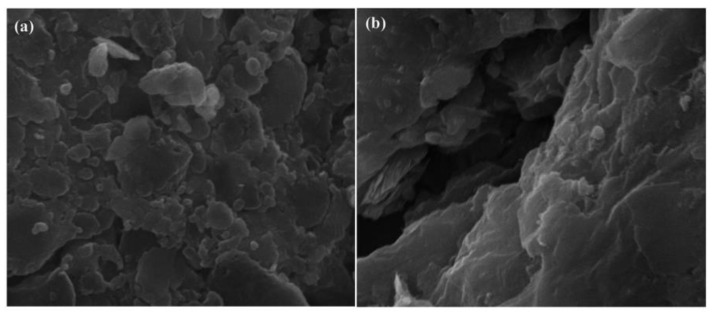
ESEM images of the oxidant-releasing material surface for the original material (before experimentation) ((**a**), 20,000×) and residual material (after experimentation) ((**b**), 10,000×) (revised from [43]).

Utilizing cost-effective and environmentally friendly loaded carriers such as starch and paraffin, KMnO_4_-releasing composites (PRCs) offer a range of benefits, including simplicity, affordability, and eco-friendliness. Liang et al. [43] focused on the development of composite slow-release materials incorporating KMnO_4_, polycaprolactone, and starch. In their study, they observed that the release rates of KMnO_4_ underwent a distinct pattern. During the initial 8 days, there was a rapid release of KMnO_4_ from the composite material. Subsequently, from day 9 to day 10, the release rate decreased at a slower pace. Notably, from day 11 to day 76, the release rate reached a relatively stable level. Over the course of the experiment, in total, 63.8% of the encapsulated KMnO_4_ was released. The ESEM images reveal that KMnO_4_ granules are deposited within the composite’s pores post-experiment, suggesting the release of KMnO_4_ from the PRCs upon contact with water (Figure 3). This finding highlights the capacity of these slow-release materials to extend the active lifetime of the oxidant, which is particularly advantageous in groundwater pollutant remediation scenarios. The flexibility and adaptability of these composite materials were further demonstrated by their ability to be fine-tuned by adjusting the ratios of KMnO_4_, starch, and PCL. This control over the release rate and lifespan of the slow-release material offers a tailored approach to environmental remediation, allowing researchers and practitioners to optimize treatment strategies for specific pollutants and conditions.

In the context of practical applications, Christenson et al. [44] explored the use of slow-release KMnO_4_ paraffin materials in the remediation of TCE contamination in a landfill site. These paraffin-based materials were designed with dimensions of 91.4 cm in length and varying diameters (5.1 cm and 7.6 cm). The results of their field-scale study were highly promising since they observed significant reductions in TCE concentrations within the treatment area. Specifically, the 7.6 cm candles achieved reductions ranging from 67% to 85%, while the 5.1 cm candles showed reductions between 10% and 66%. These findings underscore the effectiveness of using slow-release KMnO_4_ paraffin materials for the remediation of TCE contamination in low-permeability aquifers [44].

Importantly, this approach offers several distinct advantages. Firstly, it does not necessitate specialized equipment, making it accessible and feasible for a wide range of remediation projects. Secondly, it mitigates health and safety concerns associated with the use of liquid oxidants, as solid slow-release materials are more easily handled and pose fewer risks. Lastly, the longevity of this method provides a long-term solution for controlling pollutant migration during TCE remediation, reducing the need for frequent maintenance and intervention. The utilization of economical and environmentally friendly loaded carriers for KMnO_4_ slow-release materials presents a promising avenue for sustainable and effective groundwater pollutant remediation, offering both economic and environmental benefits. The adaptability of these materials allows for tailored solutions, while their practicality and long-term effectiveness make them a valuable asset in the field of environmental engineering and remediation.

#### 3.2.3. Gel-Based KMnO_4_

Gel-based controlled-release KMnO_4_ represents a versatile and effective approach for extending the active period of KMnO_4_ oxidation materials. In a study conducted by Lee et al. [45], they devised a method that involved mixing liquid resin with KMnO_4_ particles within a cylindrical mold and allowing it to crystallize at room temperature, resulting in the creation of a gel-based KMnO_4_ slow-release material. The outcomes of their research underscored the remarkable potential of this approach. One of the significant advantages of gel-based materials is their ability to control the transport permeability of MnO_4_^−^ ions in the crystalline system, which was found to be considerably smaller than that in porous sand–clay matrix systems. This property contributes to the prolonged release of active substances. Numerical simulations further revealed that these slow-release KMnO_4_ materials could sustain the release of active substances for multiple years, offering a controlled and sustained remediation solution for contaminants such as TCE. This approach is particularly well-suited for sites with limited accessibility to KMnO_4_ or low pollutant concentrations but a widespread distribution of pollutant plumes. Importantly, it addresses the issue of MnO_2_ clogging porous media, thereby promoting lateral diffusion and a more efficient mixing transport system for permanganate and pollutant plumes. Consequently, this innovative method has the potential to become a highly efficient and practical means for the on-site remediation of contaminated aquifers.

Li et al. [46] explored the use of nano-silica as a key component in gel-based KMnO_4_ slow-release materials. They mixed KMnO_4_ powder with nano-silica sol in a grinding bowl, achieving a uniform mixture of powders and sol, which was then sealed for processing. After 12 h, the release rate of various gel-based materials exceeded 85%, following pseudo-first-order kinetics. This controllable release of KMnO_4_, achieved by adjusting the ratios of KMnO_4_, the nano-silica mass fraction, and the volume of gel-based materials, highlights the adaptability and effectiveness of this method in remediating TCE pollutants. Lee et al. [47] extended the application of gel-based KMnO_4_ slow-release materials to treat groundwater contaminated with chlorinated solvents. Their research demonstrated that the gel-based material exhibited a release time of 3 days, characterized by an initial peak release of approximately 1.2 g/min, followed by a gradual decrease over 70 h. Notably, increasing the concentration of silica within the gel-based material had the practical effect of extending the release time of active KMnO_4_, offering even greater flexibility in tailoring the remediation process to specific site conditions.

Gel-based KMnO_4_ slow-release materials have emerged as a promising solution for extending the activity period of KMnO_4_ oxidation materials. They address issues related to transport permeability, clogging, and pollutant plume distribution, making them a versatile and effective option for remediating groundwater contaminated with substances like TCE and chlorinated solvents. These innovative approaches offer controlled, sustained, and adaptable remediation solutions for a variety of environmental challenges.

### 3.3. Reductant-Based Controlled-Release Materials

In the realm of remediating groundwater contamination, the utilization of reductants has gained prominence alongside oxidants. Zero-valent metals, in particular, play a pivotal role in reductive dechlorination, and their effectiveness can be enhanced through direct modification, ultimately extending their reactivity and lifespan. In situ chemical reduction (ISCR) technology, which leverages the principles of reductive dechlorination, has emerged as a widely recognized and effective method for remediating groundwater polluted with TCE [48,49].

In the chemical reduction dechlorination process, zero-valent metals like zero-valent iron (Fe^0^) and divalent iron (Fe(II)) serve as crucial reducing agents in the dechlorination reaction. Among these, zero-valent iron (ZVI) reductants stand out as essential materials for remediating TCE-contaminated groundwater. The overall reaction mechanism for remediating TCE pollution using ZVI can be succinctly represented as follows:

C_2_HCl_3_ + Fe^0^ → Hydrocarbon products + Cl^−^ + Fe_2_^+^/Fe_3_^+^ (2)

While ZVI can rapidly react with pollutants, it may not efficiently address persistent contamination plumes. One of the main drawbacks of employing metallic iron as a reductant in the degradation process is the incomplete oxidation of iron. Instead of oxidizing into Fe^3+^, metallic iron is typically oxidized into Fe^2+^ [50]. This incomplete oxidation can impact the efficiency of the remediation process, as Fe^2+^ may not be as effective in reducing organochlorine compounds. Additionally, the recovery of spent iron is a critical consideration in the application of reductant-based controlled-release materials. The retrieval and regeneration of iron from the remediation system are essential steps to minimize costs and environmental impacts. Several methods exist for the recovery of spent iron, including chemical treatments and physical separation processes. These methods should be carefully selected and integrated into the overall design of the remediation system to ensure sustainability and cost-effectiveness.

These drawbacks led to the development of innovative slow-release remediation materials. Ji et al. [50] engineered a composite material consisting of ZVI embedded in biochar, carrageenan as an encapsulation medium, and soluble starch as an organic carbon source. Their experimental results showcased outstanding slow-release performance, with this composite material achieving a TCE removal efficiency of 95.68% after 25 days—an impressive 24.69% enhancement compared to that achieved with the use of commercial remediation materials. Scanning electron microscope images revealed noteworthy changes in the microspheres, indicating decreased ZVI and soluble starch content while maintaining biochar content. Moreover, shifts in the microbial community structure pointed to the enhanced activity of functional anaerobic bacteria, particularly in dechlorination, thereby intensifying the anaerobic biodegradation of TCE. This illustrates that the slow-release composite material not only extends the release of active substances and reductant lifespan but also amplifies the effect of anaerobic bioreduction.

Nano-ZVI (nZVI) materials, characterized by their smaller size, larger specific surface area, and stronger surface reactivity compared to those of conventional ZVI reductants, have gained prominence in groundwater pollution and remediation techniques [51]. These materials are frequently employed in the treatment of chlorinated organic pollutants and heavy metals [52]. However, their properties also significantly influence their migration in the environment for the following reasons: (1) Due to their small size, nanoparticles exhibit Brownian motion during transport in water, resulting in only short-distance migration in groundwater [53]. (2) Uncoated d nZVI particles tend to aggregate easily and react with environmental pollutants, and thus the migration distance of nZVI becomes a crucial factor affecting its remediation efficiency [54]. Li et al. [55] concluded from column migration experiments that uncoated ZVI nanoparticles cannot migrate over long distances and tend to aggregate, leading to the formation of colloidal particles that clog soil pores and significantly impact their mobility in soil. (3) Microbial activity also has some influence on the migration of nZVI [56].

Sheu et al. [57] introduced a slow-release emulsion colloid material containing nZVI, vegetable oil, surfactant, molasses, lactic acid, and minerals. This material harnessed the principles of in situ chemical reduction and anaerobic biodegradation, leading to the development of an in situ biogeochemical reduction remediation technique (EHC). This approach ensured the continuous release of nano zero-valent iron for remediating TCE-contaminated groundwater. The stability of this slow-release emulsion colloid material was assessed, revealing the uniform distribution of nZVI particles (with a diameter of 100–200 nm) within the emulsion, effectively preventing agglomeration [57]. The material demonstrated the ability to continuously release active substances, with remarkable results: after 130 days, the removal efficiency of TCE with an initial concentration of 7.4 mg/L reached an impressive 99% [57]. Furthermore, the hydroxide ions generated via the oxidation of nZVI were utilized to prevent acidification, thereby reducing hydrogen sulfide production. Microbial analysis confirmed the presence of dechlorinating bacteria in the soil, suggesting that the presence of this slow-release material enhanced microbial bioactivity—an additional factor contributing to TCE dechlorination.

The utilization of reductants, particularly zero-valent metals and nZVI, has proven to be a versatile and effective approach for the remediation of groundwater contaminated with TCE. These innovative slow-release materials extend the reactivity of reductants, enhance remediation efficiency, and offer a sustainable solution to tackle persistent contamination plumes, all while considering the dynamic interplay with microbial communities and environmental factors.

### 3.4. Organic Electron Donor-Based Release Materials 

In contrast to the conventional practice of modifying the active constituents of traditional reducing agent release materials, electron donor-type reducing agent release materials introduce innovative organic compounds such as electron donors. These materials are applied in anaerobic dechlorination techniques with the primary objective of extending the duration of electron supply, thereby significantly influencing the remediation of TCE contamination in groundwater [58].

The anaerobic biodegradation of TCE hinges primarily on microbial reductive dechlorination, a process wherein chlorine atoms within chlorinated hydrocarbon molecules are substituted with hydrogen atoms under anaerobic conditions. This hydrogenation reaction, referred to as hydrogenolysis, typically follows a sequential pattern of chlorine removal. Furthermore, an alternative significant reaction pathway in chemical reduction dechlorination is β-elimination, which entails the removal of adjacent carbon atoms’ hydrogen and chlorine atoms (or two chlorine atoms) [59]. To stimulate the activity of indigenous microorganisms effectively, the provision of essential nutrients and electron donors is often imperative to maximize microbial metabolic activity. Conventionally, this has been accomplished by employing soluble sugars or readily degradable organic compounds, such as lactate or polylactate, as electron donors. However, the drawback of these commercial products lies in the need for their continuous and sustained supply into groundwater over extended periods, resulting in practical inconveniences.

To address these limitations, recent research efforts have been directed toward the development of controlled-release carbon source materials. These materials are engineered to facilitate a continuous and gradual release of organic carbon sources. In doing so, they provide sustained electron donors to support ongoing microbial activity, thereby exerting a tangible influence on the degradation of TCE in groundwater over predetermined timeframes.

#### 3.4.1. Tetrabutyl Orthosilicate (TBOS) 

Tetrabutyl orthosilicate (TBOS), also known as (CH_3_CH_2_CH_2_CH_2_O)_4_-Si, is an organosilicon compound synthesized in laboratory settings. It gained recognition approximately two decades ago for its potential in the application of anaerobic dechlorination principles to remediate TCE contamination. When subjected to hydrolysis, TBOS undergoes a transformation, yielding 1-butanol, which can be represented by the following general equation [58]:

Si(OC_4_H_9_)_4_ + 4H_2_O → 4C_4_H_9_OH + Si(OH)_4_ (3)

This equation clearly illustrates that TBOS hydrolysis leads to the production of 1-butanol. In the fermentation process of 1-butanol, the generation of butyrate and acetate salts results in the release of H_2_, which can potentially serve as an electron donor for dechlorination reactions. Consequently, tetrabutyl orthosilicate emerges as an excellent fermentation substrate capable of producing the requisite hydrogen for dehalogenation reactions.

Yu et al. [59,60] have employed controlled-release TBOS as a substrate for the microbial anaerobic dechlorination treatment of TCE-contaminated sites. In batch reactor experiments, TBOS was combined with chlorinated ethenes, yielding solutions with varying mole fractions of chlorinated ethenes (mole fraction of chlorinated ethene/(mole fraction of chlorinated ethene + mole fraction of TBOS)). Control experiments were also conducted, measuring the non-biological rate of TBOS hydrolysis and reductive dechlorination activity. The results indicated that the non-biological rate of TBOS hydrolysis is influenced by the concentration of chlorinated ethenes. At higher concentrations of dense nonaqueous phase liquid (DNAPL), the dechlorination activity of chlorinated ethenes is inhibited [60]. The accumulation of acetate and butyrate salts contributes to pH reduction, potentially diminishing the activity of dechlorinating microorganisms. Nevertheless, the measured chlorine release, directly determined in aqueous samples, corresponded to the total chlorine mass balance.

Consequently, the dehalococcoides bacterial strain can utilize the products of TBOS hydrolysis and fermentation as electron donors, facilitating the dechlorination of TCE into ethene. The use of controlled-release substrates for the anaerobic biodegradation of TCE offers several advantages, including the avoidance of repetitive or continuous injections of soluble substrates, leading to reduced operational costs. Simultaneously, it enables the distribution of chlorinated solvents to insoluble or semi-soluble substrates introduced into nearby TCE-contaminated areas, potentially mitigating the toxicity of pollutants. Therefore, TBOS holds promise as an effective anaerobic release substrate, providing controlled electron supply to enhance the sustainability of remediation efforts.

#### 3.4.2. Polyhydroxybutyrate (PHB) Organic Release Material

**Figure 4:**
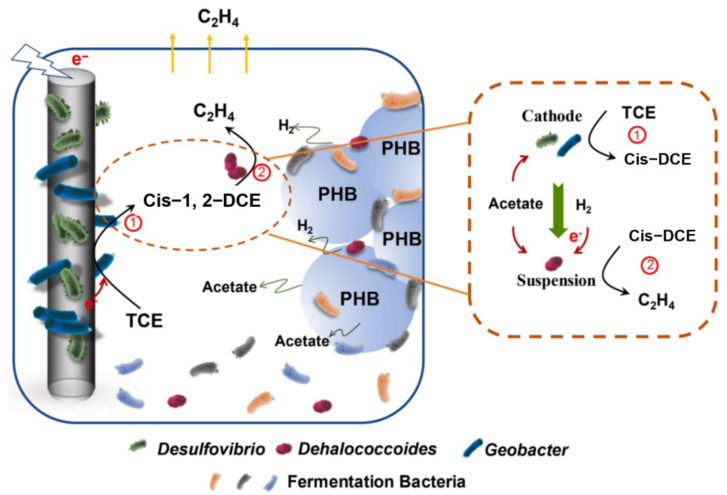
PHB facilitating bioelectrochemical dechlorination of TCE and recovery of valuable ethylene (revised from [62]).

PHB polymer materials are noteworthy for their complete biodegradability, making them ideal long-lasting release substrates capable of enhancing electron supply efficiency and effective electron provision in anaerobic dechlorination technologies. Aulenta et al. [61] pioneered a strategy that leverages fully biodegradable PHB organic release materials as a source of electron donors in the application of anaerobic dechlorination technology with bacteria serving as electron acceptors for the reductive dechlorination of TCE. During the reaction, PHB undergoes enzymatic hydrolysis, yielding 3-hydroxybutyrate, which is subsequently converted into acetate and hydrogen through β-elimination reactions. Chen et al. [62] developed a novel bioelectrochemical dechlorination system which integrated slow-release organic carbon sources and PHB. It significantly improved TCE dechlorination efficiency, resulting in a 2.23-fold higher rate constant and a 94.00 ± 1.62% recovery of valuable ethylene (Figure 4). Research findings have demonstrated that even in the presence of high TCE concentrations, reaching up to 50 mg/L, PHB can be effectively degraded into acetates and hydrogen—two crucial electron donors essential for the dechlorination of vinyl chloride. 

Baric et al. [63,64] made the noteworthy discovery that the fermentation products of PHB primarily consist of volatile fatty acids (VFAs), which play a pivotal role in sustaining and promoting bacterial growth. In their laboratory study, the synergistic use of ZVI and PHB materials within the framework of PRB technology was investigated. Notably, the fermentation of PHB not only did not impair the reactivity of ZVI but also contributed to its enhanced durability. The reducing environment created in the ZVI reaction zone further facilitated PHB fermentation. In field studies, the adoption of groundwater circulation well remediation technology was prompted by the complex geological conditions of the aquifer structure. An external processor containing ZVI and PHB was installed, with groundwater extracted from the middle and lower filtration sections through two surface-based centrifugal pumps. This extracted groundwater was then routed to the external processor for treatment, leading to the reintroduction of PHB fermentation product-rich groundwater into the aquifer. This, in turn, stimulated the growth of indigenous dechlorinating microorganisms within the groundwater permeation zone. Experimental results have unequivocally demonstrated that within the initial four months of operation, PHB fermentation products were effectively delivered to the aquifer, exerting a positive influence on biological dechlorination activity.

Haluska et al. [65] conducted experiments revealing that whether the electron donor was added in a 1:1 stoichiometric ratio to the electron acceptor or in a 10:1 ratio, the average time required for complete TCE dechlorination remained at 79 days. These experiments underscore the crucial point that augmenting the electron donor does not necessarily expedite the rate or extent of reductive dechlorination but does lead to increased costs. Therefore, for cost-effective control, it is imperative to assess the electron acceptor’s demand for the electron donor, allowing for the provision of an appropriate quantity of electrons over an extended duration. In the future, for the sake of cost optimization, a meticulous evaluation of the degree of TCE contamination and the demand for electron donors in anaerobic bioremediation technologies can significantly enhance the economic viability of organic release substrates.

## 4. Challenges and Future Outlook

**Figure 5:**
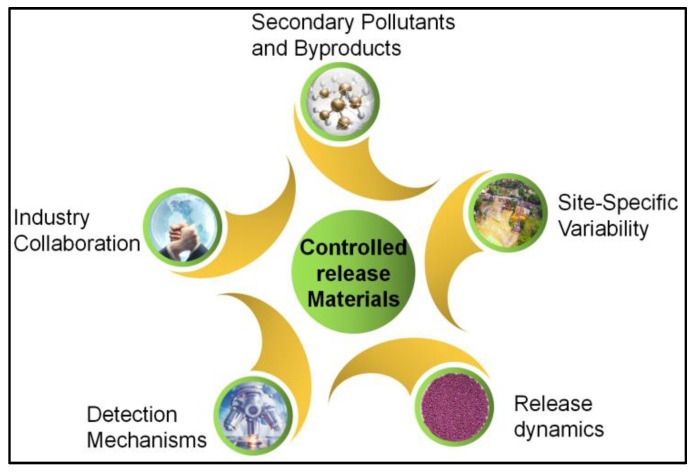
Challenges and opportunities for controlled-release materials.

While controlled-release materials have shown significant promise in the remediation of TCE contamination in groundwater, several challenges and opportunities for future research and development must be considered (Figure 5). Addressing these challenges and pursuing new directions is crucial for advancing the field and ensuring the continued effectiveness of controlled-release materials in groundwater remediation.

### 4.1. Secondary Pollutants and Byproducts

The potential formation of secondary pollutants and byproducts during the remediation process is a significant challenge. When TCE undergoes degradation, it can produce intermediate compounds or transformation products, some of which may be more toxic or persistent than the parent compound [66]. These secondary pollutants can include cis-dichloroethylene (*cis-DCE*), vinyl chloride (VC), and even more complex chlorinated compounds. The formation and fate of these byproducts must be thoroughly studied to assess their impact on the overall groundwater quality and ecosystem health. Additionally, the choice of controlled-release materials and remediation agents can influence the nature and extent of byproduct formation. For instance, the use of certain reductants or catalysts may lead to the generation of specific transformation products [67]. Researchers and practitioners must carefully consider the potential for byproduct formation and design controlled-release systems that minimize or mitigate these issues. 

Addressing the challenge of secondary pollutants and byproducts requires a comprehensive understanding of the degradation pathways and kinetics of TCE under different controlled-release material applications. Advanced analytical methods, such as mass spectrometry and high-performance liquid chromatography, should be employed to identify and quantify these transformation products [68,69]. Furthermore, research should focus on optimizing the choice of remediation agents and controlled-release materials to minimize the formation of harmful byproducts. This might involve the development of innovative encapsulation techniques or the use of alternative electron donors with fewer detrimental consequences [70]. Ultimately, balancing effective TCE degradation with the prevention of secondary contamination is a critical challenge in the field of controlled-release material-based remediation.

### 4.2. Site-Specific Variability

Groundwater contamination sites exhibit significant variability in terms of hydrogeological characteristics, contaminant concentrations, and geochemical conditions, posing a substantial challenge for the application of controlled-release materials. Hydrogeological heterogeneity, including variations in aquifer properties, permeability, and hydraulic conductivity, can greatly affect the transport of TCE and the dispersion of remediation agents within the subsurface environment [71]. The complex interplay between geological formations, groundwater flow pathways, and contaminant plume geometry necessitates the development of controlled-release materials that can adapt to diverse subsurface conditions.

Additionally, variations in contaminant concentrations and chemical speciation across different sites can impact the choice of controlled-release materials and their dosage. Higher TCE concentrations may require more aggressive remediation approaches, whereas lower concentrations might necessitate a more conservative and sustained release strategy [72]. Geochemical conditions such as pH, redox potential, and the presence of competing electron acceptors can further complicate remediation efforts [73]. 

Therefore, addressing the challenge of site-specific variability in controlled-release materials for TCE remediation requires the development of adaptable and customizable solutions that can account for the diverse subsurface conditions encountered in different groundwater contamination scenarios. Researchers and practitioners must work together to refine existing technologies and develop innovative materials and delivery systems capable of responding to the unique challenges posed by each contaminated site. Furthermore, advanced site characterization techniques, including geophysical surveys and groundwater modeling, can help provide a better understanding of subsurface heterogeneity and aid in the selection and design of controlled-release materials tailored to specific sites [74]. Collaboration among multidisciplinary teams of hydrogeologists, chemists, engineers, and environmental scientists is crucial to addressing this challenge effectively. Additionally, the integration of real-time monitoring and adaptive control strategies into remediation systems can enhance the ability to respond to changing subsurface conditions, ultimately improving the success rates of controlled-release material applications in the field [75,76].

### 4.3. Release Dynamics

One of the critical challenges associated with controlled-release materials for TCE remediation lies in achieving optimal release dynamics. The rate at which remediation agents are released from these materials significantly affects the overall efficiency of the remediation process. Too rapid a release may result in a quick depletion of the active agent, leaving the site under-remediated, while excessively slow release kinetics may prolong the remediation period and hinder its cost-effectiveness. Achieving the right balance in release dynamics is complicated by the heterogeneity of subsurface conditions, which can lead to non-uniform agent distribution and release rates [77]. Furthermore, the behavior of controlled-release materials may vary depending on the type of encapsulation, matrix, or delivery system employed. For example, encapsulated materials may release their remediation agents differently compared to gel-based systems, and the choice of controlled-release mechanisms, such as diffusion or degradation, can further influence release kinetics [78]. 

To address the challenge of release dynamics, researchers must investigate and optimize controlled-release materials and systems to ensure their effectiveness across various subsurface conditions. Advanced modeling approaches, such as numerical simulations and mathematical models, can be employed to predict and simulate the release behavior of these materials under different scenarios [79]. Additionally, the capabilities for the direct and real-time monitoring of the release and dynamics of drugs in living systems may help realize optimal remediation conditions [80]. Collaborative efforts between material scientists, hydrogeologists, and engineers are essential to design controlled-release materials that can provide precise, adaptable, and efficient release dynamics for TCE remediation.

### 4.4. Detection Mechanisms

Developing effective detection mechanisms for released active substances is a critical aspect of controlled-release-material-based TCE remediation. Reliable methods for monitoring the presence and concentration of active agents in groundwater are essential for assessing the performance of controlled-release materials [81]. Traditional groundwater monitoring techniques, such as grab sampling and laboratory analysis, may not be suitable for real-time or in situ assessments due to their time-consuming nature, limited spatial coverage, and the potential for underrepresenting the spatial and temporal variability of contaminants in the subsurface [82]. Therefore, the challenge lies in designing innovative monitoring systems that can provide timely and accurate data on the fate and transport of remediation agents.

To address this challenge, emerging technologies offer promising solutions. In situ sensors and monitoring networks have gained traction for continuously measuring the concentration of active agents in the subsurface [83]. These sensors can offer real-time data, enabling researchers and practitioners to track the progress of remediation, assess the effectiveness of controlled-release materials, and make timely adjustments to remediation strategies if needed. Advanced analytical techniques, such as spectroscopy, immunoassays, and electrochemical sensors, can provide high sensitivity and specificity in detecting the trace concentrations of active substances, even in complex groundwater matrices [84]. Incorporating these technologies into monitoring systems can significantly enhance our ability to quantify the presence of active agents accurately. Moreover, remote sensing technologies and geophysical methods, such as electrical resistivity tomography and ground-penetrating radar, have been explored to provide valuable insights into the spatial distribution and movement of contaminants and remediation agents in the subsurface [85]. Collaborative efforts among researchers, sensor manufacturers, and environmental agencies are crucial for advancing the state-of-the-art in groundwater monitoring technology and ensuring that these systems can reliably track the fate of active agents during TCE remediation processes.

### 4.5. Industry Collaboration 

One significant challenge in advancing the use of controlled-release materials for TCE remediation lies in fostering collaboration between the research and development (R&D) community and industry stakeholders. While research institutions and universities are at the forefront of developing innovative materials and technologies, the successful deployment and scaling up of controlled-release materials for practical field applications often require the active involvement of industry partners. However, bridging the gap between academia and industry in the environmental remediation sector can be challenging due to various factors, including differences in objectives, priorities, and timelines [86].

The future outlook for addressing the challenge of industry collaboration in the context of controlled-release material-based TCE remediation is promising but requires concerted efforts from all stakeholders. To facilitate collaboration, researchers and academic institutions should actively engage with industry partners early in the research and development process. This could involve establishing industry advisory boards, participating in joint research projects, and conducting pilot-scale studies in collaboration with industry experts [87]. Such partnerships can help align research efforts with industry needs, ensuring that controlled-release materials are not only effective but also practical and economically viable for large-scale implementation. Furthermore, industry collaboration can lead to the development of standardized protocols and guidelines for the use of controlled-release materials in TCE remediation, which can help streamline regulatory approvals and facilitate broader adoption [88]. Additionally, collaborations can support technology transfer and capacity building, enabling industry stakeholders to adopt and implement innovative remediation approaches more effectively. Ultimately, fostering a culture of collaboration between academia and industry is essential for realizing the full potential of controlled-release materials in addressing TCE contamination challenges and advancing sustainable groundwater remediation practices.

## 5. Conclusions

The TCE contamination of groundwater poses a significant environmental challenge. Traditional remediation methods, while effective to some extent, have limitations related to cost, energy consumption, and incomplete TCE removal. In response to these challenges, controlled-release materials have emerged as a promising approach for TCE remediation. Controlled-release materials slowly release remediation agents into groundwater, promoting sustained TCE degradation while minimizing the drawbacks associated with traditional methods. However, several challenges must be addressed to fully realize their potential.

The formation of secondary pollutants during TCE degradation requires advanced analytical techniques and careful material selection. In future, it can be combined with subsequent biodegradation to improve the remediation efficiency. Site-specific variability in hydrogeological conditions highlights the need for adaptable solutions and multidisciplinary collaboration. Achieving optimal release dynamics necessitates modeling and ongoing material development, and effective detection mechanisms are essential for accurate progress monitoring. Furthermore, fostering collaboration between academia and industry is crucial for the practical application of controlled-release materials. Early engagement, joint research projects, and technology transfer are key strategies with which to bridge the gap between research and practical solutions.

In conclusion, controlled-release materials offer promise for addressing TCE contamination in groundwater, providing a more sustainable and efficient approach to remediation. By addressing the challenges outlined and pursuing the recommended strategies, this field can advance, contributing to improved groundwater cleanup efforts worldwide.

## Figures and Tables

**Table 1:** Traditional remediation methods and their mechanisms, advantages, and limitations.

Traditional Remediation Methods	Mechanisms	Advantages	Limitations
Pump and Treat	Groundwater extraction and treatment	- Established method - Effective in reducing TCE concentrations - Commonly used - Proven technology	- Energy-intensive - May not address low-permeability zones - Prolonged treatment periods - Potential for incomplete remediation
Air Sparging	Injection of air for volatilization and	- Enhances volatilization and biodegradation of TCE - Widely applied - Potential for contaminant release into atmosphere	- Ineffective for dissolved-phase TCE - Challenges in controlling injected air
Chemical Oxidation	In situ chemical transformation of TCE; using reagents (e.g., permanganate)	- TCE transformation into less harmful compounds - Potential for efficient treatment - Proven effectiveness	- Requires controlled injection Risk of excessive reagent use - Formation of harmful byproducts
Bioremediation	Microbial degradation of TCE and transformation into less toxic substances	- Environmentally friendly - Cost-effective - Potential for natural attenuation	- Slow treatment rates - Influenced by various factors (e.g., temperature) - Limited microbial activity in some areas
Barrier Walls and Containment	Physical containment of TCE plumes	- Effective in isolating and controlling plumes - Can prevent further spreading of contamination - Suitable for specific scenarios	- Expensive - May not provide permanent solution - Limited effectiveness in dynamic hydrogeological sites

## Data Availability

No new data were created.
